# Pan‐cancer analysis of alternative splicing regulator heterogeneous nuclear ribonucleoproteins (hnRNPs) family and their prognostic potential

**DOI:** 10.1111/jcmm.15558

**Published:** 2020-09-11

**Authors:** Hao Li, Jingwei Liu, Shixuan Shen, Di Dai, Shitong Cheng, Xiaolong Dong, Liping Sun, Xiaolin Guo

**Affiliations:** ^1^ Department of Laboratory Medicine The First Affiliated Hospital of China Medical University Shenyang China; ^2^ Department of Anorectal Surgery the First Affiliated Hospital of China Medical University Shenyang China; ^3^ Tumor Etiology and Screening Department of Cancer Institute and General Surgery Key Laboratory of Cancer Etiology and Prevention The First Hospital of China Medical University China Medical University Shenyang China

**Keywords:** alternative splicing, hnRNPs, pan‐cancer

## Abstract

As the most critical alternative splicing regulator, heterogeneous nuclear ribonucleoproteins (hnRNPs) have been reported to be implicated in various aspects of cancer. However, the comprehensive understanding of hnRNPs in cancer is still lacking. The molecular alterations and clinical relevance of hnRNP genes were systematically analysed in 33 cancer types based on next‐generation sequence data. The expression, mutation, copy number variation, functional pathways, immune cell correlations and prognostic value of hnRNPs were investigated across different cancer types. HNRNPA1 and HNRNPAB were highly expressed in most tumours. HNRNPM, HNRNPUL1, and HNRNPL showed high mutation frequencies, and most hnRNP genes were frequently mutated in uterine corpus endometrial carcinoma (UCEC). HNRNPA2B1 showed widespread copy number amplification across various cancer types. HNRNPs participated in cancer‐related pathways including protein secretion, mitotic spindle, G2/M checkpoint, DNA repair, IL6/JAK/STAT3 signal and coagulation, of which hnRNP genes of HNRNPF, HNRNPH2, HNRNPU and HNRNPUL1 are more likely to be implicated. Significant correlation of hnRNP genes with T help cells, NK cells, CD8 positive T cells and neutrophils was identified. Most hnRNPs were associated with worse survival of adrenocortical carcinoma (ACC), liver hepatocellular carcinoma (LIHC) and lung adenocarcinoma (LUAD), whereas hnRNPs predicted better prognosis in kidney renal clear cell carcinoma (KIRC) and thymoma (THYM). The prognosis analysis of KIRC suggested that hnRNPs gene cluster was significantly associated with overall survival (HR = 0.5, 95% CI = 0.35‐0.73, *P* = 0.003). These findings provide novel evidence for further investigation of hnRNPs in the development and therapy of cancer in the future.

## 1 INTRODUCTION

RNA splicing procedure removes introns and combines exons of pre‐mature mRNA, which is essential for cellular homoeostasis, functional regulation, tissue development and species diversity.^1^, ^2^ Almost each transcript derived from human genes undergoes diverse patterns of alternative splicing (AS) including exclusion or inclusion of ‘‘cassette’’ exons, changes of AS sites, intron retentions, alternative promoter or terminator, and mutually exclusive exons.^3^, ^4^ Alternative splicing of pre‐mRNA is responsible various aspects of biological processes and aberrant AS contribute to a series of disorders even cancer.^5^, ^6^ Emerging evidence has demonstrated that cancer cells hijack and alter AS process, thereby facilitating its growth and metastasis.^7^, ^8^

As the most critical alternative splicing regulator, heterogeneous nuclear ribonucleoproteins (hnRNPs) family are responsible for the maturation of pre‐mRNAs into functional mRNAs as well as the stabilization of mRNA translocation.^9^, ^10^ Through the RNA binding domains (RBDs), hnRNPs accomplish the recognition of specific RNA sequences and control various biological processes of RNA function and metabolism.^11^, ^12^ Mechanistically, hnRNPs constitute mRNA‐protein 40S core complex via binding to RNA elements including exon and intron splicing regulators, which precisely control the alternative splicing of pre‐mRNAs.^13^ Until now, approximately twenty key members of hnRNPs family have been identified including hnRNP A‐U, which share common characteristics but differ in biological properties.^14^

Emerging evidence has suggested close relationship between hnRNPs and multiple malignant behaviours of cancer.^15^ For instance, hnRNP A1 modulates the alternative splicing of CDK2, thereby contributing to oral squamous cell carcinoma by altering cell cycle progression.^16^ In pancreas cancer, hnRNP E1 cancer cell metastasis via controlling the alternative splicing of integrin β1, a membrane receptor involved in cell adhesion, immune response and metastatic diffusion of cancer cells.^17^ Studies have suggested that hnRNP A1, A2/B1 and K bind to the promoter of tumour suppressor Annexin‐A7, which alters Annexin‐A7 splicing patterns and leads to prostate cancer.^18^ In addition, hnRNP L has been found to regulate VEGFA mRNA translation and induce apoptosis of cancer cells, thereby inhibiting the development of cancer.^19^

In spite of the current reports indicating the significant contribution of hnRNPs in carcinogenesis, our knowledge of the specific implication concerning hnRNPs still remains limited. Considering the increasing essential role of hnRNPs in cancer, it is of great interest to unravel the whole landscape of expression, mutation and copy number variation of alternative splicing regulator hnRNPs family as well as their prognostic potential. Through analysing multiple levels of data from The Cancer Genome Atlas (TCGA) including 33 types of cancers, we described the specific implication of alternative splicing regulator hnRNPs in various cancers in this study. It is anticipated that the comprehensive pan‐cancer analysis could shed light on the way alternative splicing lead to cancer.

## 2 MATERIALS AND METHODS

### 2.1 Collection of hnRNP genes

We collected 22 hnRNP genes from recently published review papers. All these gene symbols were converted into Ensemble gene IDs and HGNC symbols by manually curated from GeneCards (https://www.genecards.org/).

### 2.2 Genome‐wide omics data across 33 cancer types from next‐generation sequence data

The results in our analysis were based upon omics datasets generated by TCGA Research Network (http://cancergenome.nih.gov/). We totally analysed 33 different TCGA projects, and each project represented a specific cancer type, including KIRC, kidney renal clear cell carcinoma; KIRP, kidney renal papillary cell carcinoma; KICH, kidney chromophobe; LGG, brain lower‐grade glioma; GBM, glioblastoma multiforme; BRCA, breast cancer; LUSC, lung squamous cell carcinoma; LUAD, lung adenocarcinoma; READ, rectum adenocarcinoma; COAD, colon adenocarcinoma; UCS, uterine carcinosarcoma; UCEC, uterine corpus endometrial carcinoma; OV, ovarian serous cystadenocarcinoma; HNSC, head and neck squamous carcinoma; THCA, thyroid carcinoma; PRAD, prostate adenocarcinoma; STAD, stomach adenocarcinoma; SKCM, skin cutaneous melanoma; BLCA, bladder urothelial carcinoma; LIHC, liver hepatocellular carcinoma; CESC, cervical squamous cell carcinoma and endocervical adenocarcinoma; ACC, adrenocortical carcinoma; PCPG, pheochromocytoma and paraganglioma; SARC, sarcoma; LAML, acute myeloid leukaemia; PAAD, pancreatic adenocarcinoma; ESCA, oesophageal carcinoma; TGCT, testicular germ cell tumours; THYM, thymoma; MESO, mesothelioma; UVM, uveal melanoma; DLBC, lymphoid neoplasm diffuse large B‐cell lymphoma; CHOL, cholangiocarcinoma. All of the TCGA data including TPM (Transcripts Per Kilobase Million) expression, copy number variation, mutation and clinical information (survival status, stages, grades, survival time) were download from UCSC XENA (https://xenabrowser.net/).

### 2.3 Identification of differentially expressed genes

To identify the alternation of gene expression in each cancer type, we used the Deseq2 package in R to identify differentially expressed genes. Genes with adjusted *P*‐values < 0.05 and at least twofold changes in expression were identified as differentially expressed genes in each cancer type.

### 2.4 Protein‐wide omics data across pan‐cancer from protein expression data

The protein expression data of hnRNP genes were obtained from 'The Human Protein Atlas' database (https://www.proteinatlas.org/). We totally analysed 20 cancer types on hnRNP genes protein expression, including BRCA (breast cancer), carcinoid (carcinoid), CECA (cervical cancer), COCA (colorectal cancer), glioma (glioma), HNSC (head and neck cancer), LIHC (liver cancer), LUCA (lung cancer), lymphoma (lymphoma), melanoma (melanoma), OV (ovarian cancer), PACA (pancreatic cancer), RACA (renal cancer), SKCA (skin cancer), STCA (stomach cancer), TECA (testis cancer), THCA (thyroid cancer), URCA (urothelial cancer), ENCA (endometrial cancer) and PRCA (prostate cancer).

### 2.5 Genome‐wide mutation data across pan‐cancer cell lines from CCLE datasets

Mutation frequency of hnRNP family genes in pan‐cancer cell lines were obtained from Cancer Cell Line Encyclopedia (CCLE) datasets (https://portals.broadinstitute.org/ccle).

### 2.6 Oncogenic pathway activity across cancer types

In order to calculate the activity of cancer hallmark‐related pathways, the TPM gene expression was subjected to gene set variation analysis (GSVA), which is a non‐parametric unsupervised method for estimating variation of gene set enrichment through the samples of an expression dataset. To identify the hnRNP genes that were correlated with activation or inhibition of certain pathway, we calculated the Pearson correlation coefficient (PCC) between expression of hnRNP genes and pathway activity. The regulator‐pathway pairs with |PCC|>0.3 and adjusted *P*‐value < 0.05 were identified as significantly correlated hnRNP genes.

### 2.7 Correlation of hnRNP genes with immune‐related genes

The major immune cells related genes were shown in Table S1. In order to explore the correlation between hnRNP genes and immune‐related genes, we calculated the Spearman correlation coefficient (SCC) between expression of hnRNP genes and immune‐related genes. The regulator‐pathway pairs with |PCC|>0.3 and adjusted *P*‐value < 0.05 were identified as significantly correlated hnRNP genes.

### 2.8 Clinical significance of hnRNP genes

To explore whether the expression of hnRNP genes was associated with patient survival, we divided all the patients into two groups based on the median expression of each hnRNP gene. The log‐rank test was used to test the different survival rates between the two groups. The *P*‐values < 0.05 were considered as statistical significance.

## 3 RESULTS

### 3.1 Expression profile of hnRNP genes across different cancer types

**FIGURE 1:**
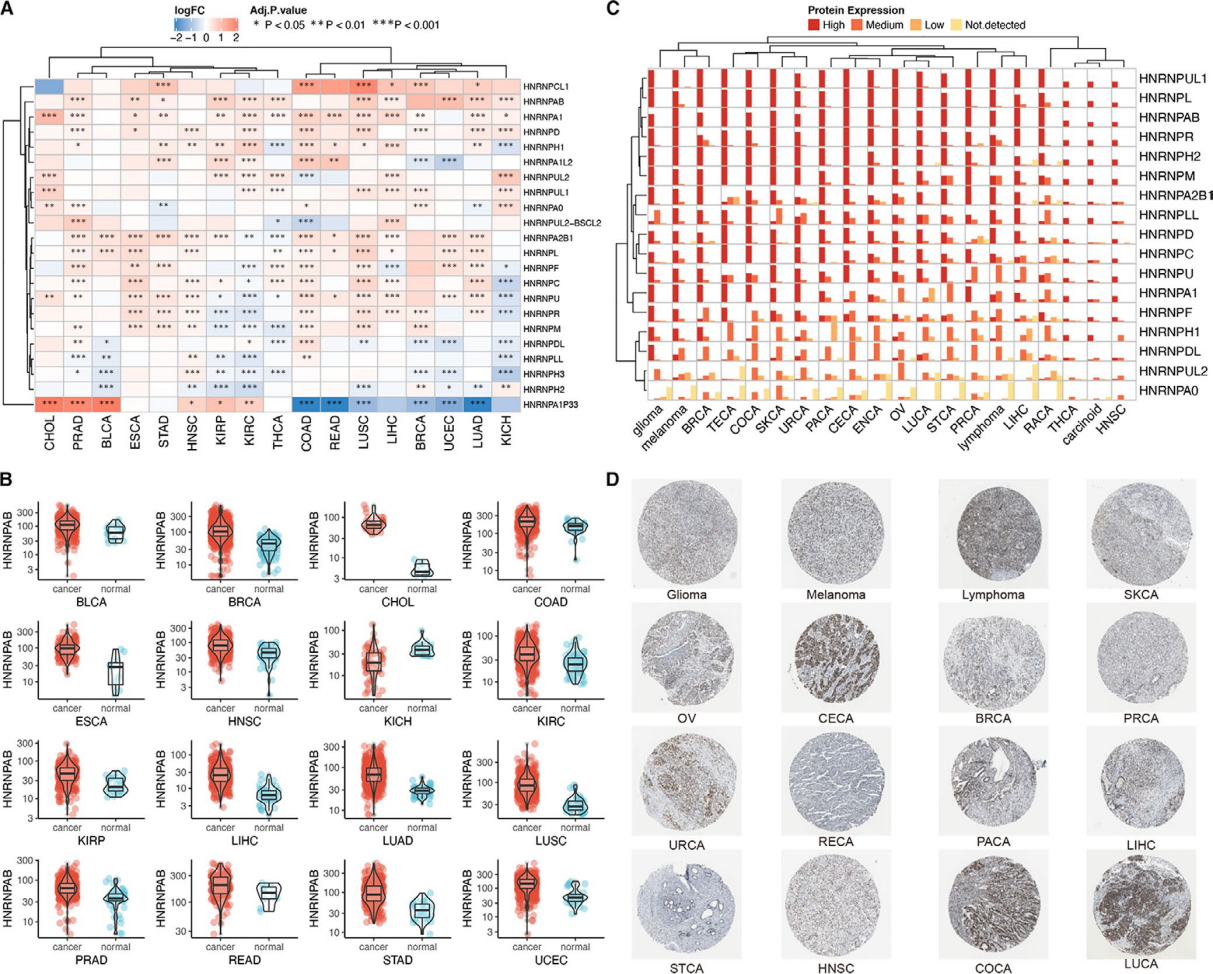
Expression profile of hnRNP genes across different cancer types. (A) Expression of hnRNP genes in different cancer and normal samples. The colour in heat map represents the log2 fold change value between cancer and normal. The blue colour represents the low expression in cancer, whereas the red colour represents the high expression in cancer. The * sign represents degree of statistical significance. (B) HNRNPAB expression in 16 types of cancers between cancer and normal tissues. (C) hnRNP protein expression across various cancer types. Each gene expression in one cancer was divided into four groups of high expression, medium expression, low expression and not detected. (D) HNRNPD gene protein expression in 16 cancer types based on immunohischemistry staining results from 'The Human Protein Atlas' database

A total of 22 hnRNP genes were identified after searching the published review papers, the information of which was summarized in Table S1. Using the count data of TCGA, we described the differential expression of these genes across different cancer types. As shown in Figure 1A, hnRNP genes demonstrated heterogeneous distributions in different cancer types: HNRNPA1 and HNRNPAB were highly expressed in most tumours; HNRNPA1P33 expression was increased in COAD, READ and LUAD whereas decreased in CHOL, PRAD and BLCA. The detailed LogFC changes were listed in Table S2. Next, we visualized the differential expression of HNRNPAB in each cancer (Figure 1B). Based on the immunohischemistry results of Protein Atlas database, we showed the protein expression of hnRNP genes in various cancer types (Figure 1C). In addition, immunohischemistry results of HNRNPD based on 'The Human Protein Atlas' database representing the protein expression was shown in Figure 1D.

### 3.2 Pan‐cancer genetic alternations of hnRNP genes

**FIGURE 2:**
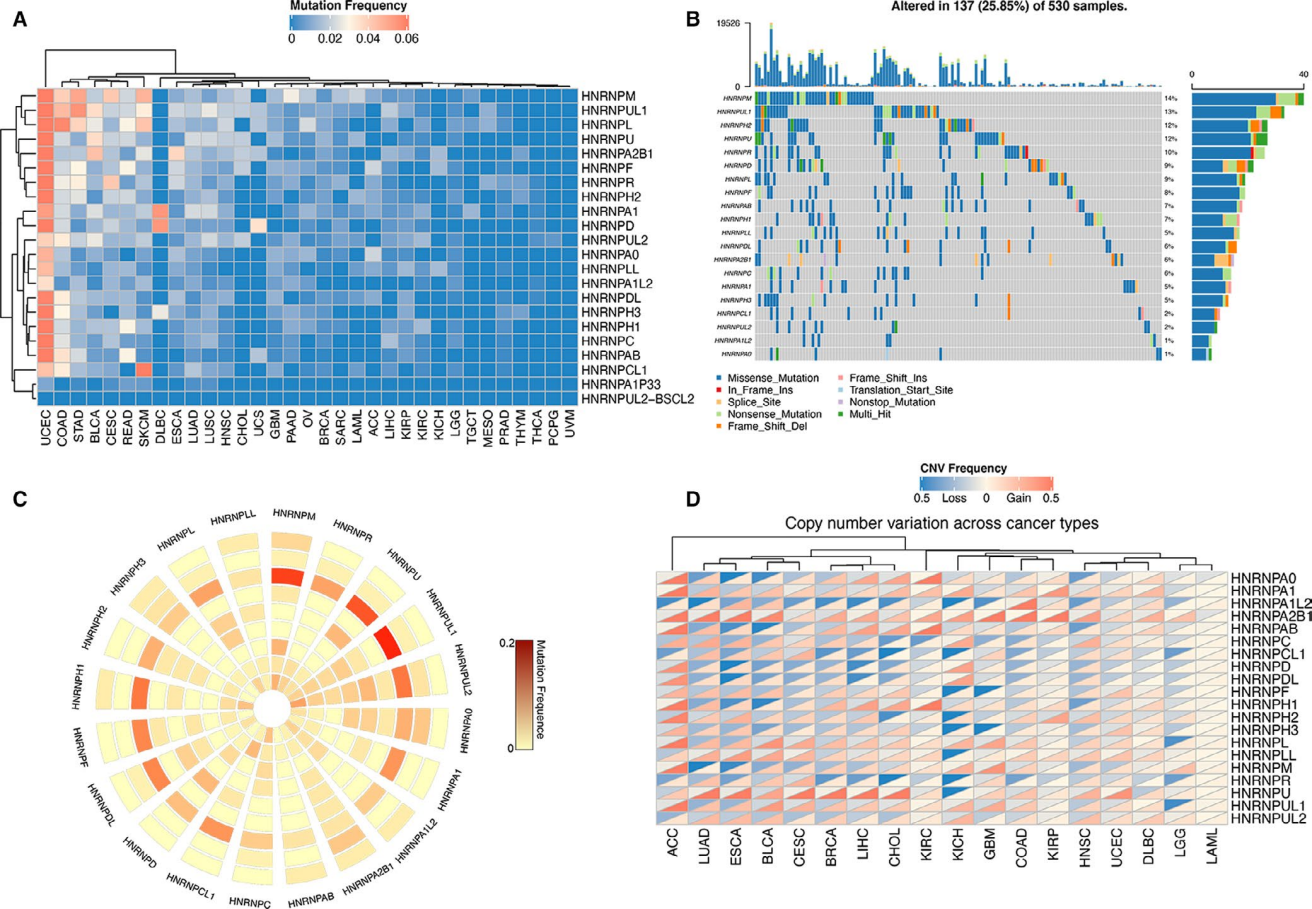
Pan‐cancer genetic alternations of hnRNP genes. (A) Pan‐cancer mutation frequency of hnRNP genes. (B) Oncoplot for hnRNP genes in UCEC. HNRNPM showed the most frequent mutation in UCEC. (C) The mutation frequency of hnRNP genes across common cancer cell lines. Each circle from the outside to the inside represents a type of tumour cell line (breast, gastric, colorectal, kidney, lung, bone, ovary, skin, fibroblast and liver). (D) The copy number variations frequency of hnRNP genes in different cancers

The mutation frequency of hnRNP genes were analysed, and the results indicated that most hnRNP genes were frequently mutated in UCEC (Figure 2A). The overall average mutation frequency ranged from 0% to 14.9%, and hnRNP genes including HNRNPM, HNRNPUL1, HNRNPL showed relatively high mutation frequencies. Several cancers such as THCA, PCPG and UVM demonstrated rare hnRNP gene mutations. In order to show more detailed information about hnRNP mutation, we then visualized the mutation details of hnRNP genes in UCEC by oncoplot (Figure 2B). Besides, CCLE database was used to demonstrate the mutation status of hnRNP genes in various human cancer cell lines (Figure 2C). The results indicated that colorectal cancer and lung cancer cell lines suggested frequent mutations of most hnRNP genes. In addition, the copy number variations of hnRNP genes were also investigated across different cancer types (Figure 2D): HNRNPA2B1 gene showed widespread copy number amplification across various cancer types whereas almost no CNV was detected in LAML.

### 3.3 Association of hnRNPs with cancer‐related pathways and immune status

**FIGURE 3:**
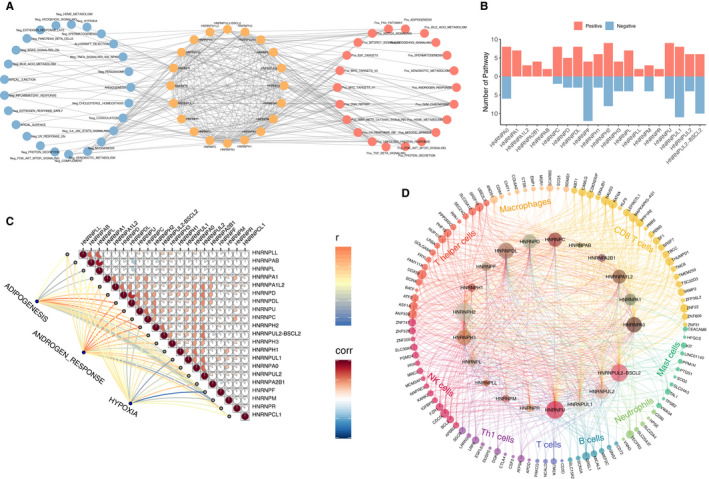
Association of hnRNPs with cancer‐related pathways and immune status. (A) Network diagram demonstrating the correlation between hnRNP genes and cancer‐related pathways. Blue node represents the negative correlation pathways, whereas red nodes represent the positive pathways. (B) The number of correlated pathways in each individual hnRNP genes. (C) Correlation between the expression of different hnRNP genes and the correlation between the three tumour‐associated pathways and individual hnRNP genes. (D) Correlation between hnRNP genes and immune cells infiltration. The genes in the outer circle represent genes within individual immune cells. Inner circles are formed by hnRNP genes. The size of each gene represents the number of connections

In order to elucidate the molecular implication of hnRNPs in carcinogenesis, the relation of hnRNPs with cancer‐related pathways was analysed and visualized in Figure 3A. The findings suggested that hnRNP expressions significantly correlated with the activation or suppression of various oncogenic pathways. It could be concluded that hnRNP genes mainly participated in cancer‐related pathways including protein secretion, mitotic spindle, G2/M checkpoint, DNA repair, IL6/JAK/STAT3 signal and coagulation. At the same time, the numbers of the correlated pathways of each gene were summarized, of which hnRNP genes of HNRNPF, HNRNPH2, HNRNPU and HNRNPUL1 are more likely to be implicated in oncogenic processes (Figure 3B). As pathways of adipogenesis, androgen response and hypoxia showed different correlations with diverse hnRNP genes, we summarized the correlations among different hnRNP genes as well as the specific correlation with adipogenesis, androgen response and hypoxia in Figure 3C. We found that hnRNPs might work together in carcinogenesis as significant correlations were detected such as HNRNPL‐HNRNPAB (*r* = 0.83), HNRNPUL2‐HNRNPA0 (*r* = 0.58) and HNRNPAB‐HNRNPLL (*r* = 0.57). At last, the effect of hnRNP genes on immune cell infiltration was shown in Figure 3D. The most relevant immune cells included T help cells, NK cells, CD8 positive T cells and neutrophils. HNRNPH2, HNRNPU, HNRNPDL and HNRNPA0 all demonstrated significant correlation with immune cell infiltration.

### 3.4 Prognostic significance of hnRNP genes

**FIGURE 4:**
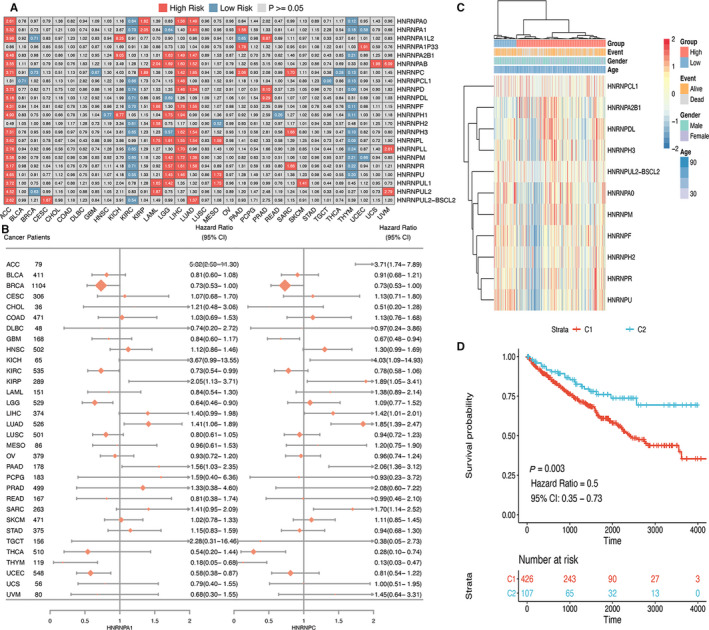
Prognostic significance of hnRNP genes. (A) Summary of the correlation between expression of hnRNP genes and survival of different cancers. Red colour represents high risk of death, whereas blue colour represents low risk of death. (B) Forest plot for the prognostic analysis of HNRNPA1 and HNRNPC across various cancer types. (C) Heat map showing the clustering for KIRC patients based on the expression of hnRNP genes. (D) Survival analysis for cluster group based on hnRNP genes in KIRC

The prognostic significance of hnRNP genes in different cancer types was analysed by Cox regression (Figure 4A). In cancers including ACC, LIHC and LUAD, most hnRNPs were associated with worse survival of cancer patients. In contrast, hnRNPs predicted better prognosis in cancers such as KIRC and THYM. In addition, certain hnRNP gene might exert obvious different prognostic effect across various cancer types. For instance, HNRNPA1 and HNRNPC showed different prognostic association in diverse cancer types, which were therefore shown by forest plot to illustrate the specific predictive effect in diverse types of cancers (Figure 4B). As many hnRNP genes demonstrated influence on KIRC prognosis, we performed clustering analysis of prognosis‐related hnRNP genes (Figure 4C). The prognosis analysis of the cluster C1 and C2 suggested that C2 cluster was significantly associated with better survival compared with C1 cluster (HR = 0.50, 95% CI = 0.35‐0.73, *P* = .003), indicating the promising potential of hnRNP genes in the prediction of cancer prognosis (Figure 4D).

## 4 DISCUSSION

In order to clarify the critical role of alternative splicing regulator heterogeneous nuclear ribonucleoproteins family across various types of cancer, we comprehensively analysed the core genes which belong to hnRNPs family. Based on multiple levels of data from TCGA, genomic and transcriptomic landscape of key hnRNPs family genes was investigated by pan‐cancer analysis. The results suggested that hnRNPs were differentially expressed in certain cancers and corresponding controls, which also correlated with prognosis of patients. The identified correlation between hnRNPs with multiple cancer‐related pathways suggested close implication of hnRNPs in the development of various types of cancers.

By comprehensively analysing the transcriptional data of 22 core hnRNP genes in TCGA, we describe the expression landscape of hnRNP genes across different cancer types. Heterogeneous distributions of hnRNP genes were observed in different cancer types: HNRNPA1 and HNRNPAB were highly expressed in most tumours. It has been reported that hnRNPA1 was highly expressed in gastric cancer tissues, which promote proliferation, migration and EMT of gastric cancer cells.^20^ In lung cancer, knockdown of HNRNPA1 suppressed the viability and growth as well as induced cell cycle arrest of lung cancer cells.^21^ The results of previous studies and our analysis all suggested the critical role of HNRNPA1 in the initiation and development of different types of cancers. Besides, HNRNPAB overexpression has been found in metastatic cells or cancer tissues in hepatocellular carcinoma patients, which lead to EMT and metastasis of hepatocellular carcinoma cells in vivo.^22^ The oncogenic effect of HNRNPA1 and HNRNPAB is of great interest to understand the underlying mechanisms of alternative splicing in carcinogenesis, which might provide novel insights into anti‐tumour therapy. Moreover, HNRNPA1P33 expression was increased in COAD, READ and LUAD whereas decreased in CHOL, PRAD and BLCA, which indicated that hnRNPs might exert different functions in diverse kinds of tumours.

Pan‐cancer genetic alternations of hnRNP genes indicated that the overall average mutation frequency ranged from 0% to 14.9%, and hnRNP genes including HNRNPM, HNRNPUL1, HNRNPL showed high mutation frequencies. The critical role of HNRNPM in the development and metastasis has been investigated in colon cancer,^23^ prostate cancer^24^ and breast cancer.^25^, ^26^ Importantly, next‐generation sequencing has suggested HNRNPL as a key regulator of prostate cancer via modulating the alternative splicing of multiple RNAs such as the core oncogene androgen receptor.^27^ It is worth noting that most hnRNP genes were frequently mutated in UCEC, a certain type of cancer with high global mutation burden.^28^ Several cancers such as THCA, PCPG and UVM demonstrated rare hnRNP gene mutations. Besides, human cancer cell lines analysis based on CCLE demonstrated that colorectal cancer and lung cancer cell lines possess frequent mutations of most hnRNP genes. Future investigations concerning the mutations of hnRNP genes in lung cancer and colorectal cancer might reveal critical evidence of contribution of hnRNPs in the development of cancer. In addition, the copy number variations investigation revealed that HNRNPA2B1 gene showed widespread copy number amplification across various cancer types whereas almost no CNV was detected in LAML.

The correlation analysis of hnRNPs with cancer‐related pathways suggested that hnRNPs significantly contributed to the activation or suppression of various oncogenic pathways including protein secretion, mitotic spindle, G2/M checkpoint, DNA repair, IL6/JAK/STAT3 signal and coagulation. Different hnRNPs were found to be associated with distinct cancer pathway alterations, suggesting different functional effects of hnRNPs within the same alternative splicing regulator family. HNRNPA1 was significantly associated with pathways including DNA repair, G2/M checkpoint, E2F targets and myc targets. HNRNPAB showed correlation with G2/M checkpoint and wnt‐β‐catenin pathways. In addition, hnRNP genes of HNRNPF, HNRNPH2, HNRNPU and HNRNPUL1 are more likely to be implicated in oncogenic processes. Previously, HNRNPU has been reported to facilitate chromatin looping and p300‐mediated transactivation of transcription factor early growth response 1, thus promoting cancer progression.^29^ Furthermore, we also found that hnRNPs might work together in carcinogenesis as significant correlations were detected such as HNRNPL‐HNRNPAB, HNRNPUL2‐HNRNPA0 and HNRNPAB‐HNRNPLL. As for immune cell infiltrations, the most relevant immune cells of hnRNPs included T help cells, NK cells, CD8 positive T cells and neutrophils. Genes of HNRNPH2, HNRNPU, HNRNPDL and HNRNPA0 all demonstrated significant correlation with immune cell infiltration. HNRNPU has been found to interact with NF‐κB‐responsive Long Non‐coding RNA FIRRE to modulate the mRNAs of certain inflammatory genes in innate immune system.^30^ The close relation between alternative splicing regulator hnRNPs and immune system might offer new idea for future studies on immune therapy against cancer.

Pan‐cancer prognostic analysis of hnRNP genes suggested that most hnRNPs were associated with worse survival of cancer patients in cancers including ACC, LIHC and LUAD. However, hnRNPs predicted better prognosis in cancers such as KIRC and THYM. In addition, HNRNPA1 predicted worse prognosis of cancers including ACC, KIRP, LUAD and PAAD but was associated with better survival in cancers of KIRC, LGG, THYM and UCEC. These results suggested that HNRNPA1 might exert obviously different prognostic effect across various cancer types. Previously, high HNRNPUL2 expression has been reported to predict poor survival of multiple cancers.^31^ Significant association of HNRNPH expression and prognosis of colorectal cancer patients has been suggested by tissue microarray.^32^ Oral squamous cell carcinoma patients with increased HNRNPD expression significantly correlated with shorter recurrence‐free survival.^33^ These findings indicated that hnRNPs were closely implicated in the prognosis of various cancers. As many hnRNP genes demonstrated influence on KIRC prognosis, we further performed clustering analysis of prognosis‐related hnRNP genes. The prognosis analysis of the cluster C1 and C2 suggested that C2 cluster was significantly associated with better survival compared with C1 cluster, indicating that hnRNP genes might be used as a prognostic predictor of cancer in the future.

## 5 CONCLUSION

In summary, our study systematically demonstrated the expression, mutation, copy number variation, functional pathways and prognostic value of alternative splicing regulator hnRNPs across a series of cancers. The expressions of hnRNPs suggested significant association with oncogenic pathways including protein secretion, mitotic spindle, G2/M checkpoint, DNA repair, IL6/JAK/STAT3 signal and showed correlation with immune regulations of T help cells, NK cells, CD8 positive T cells and neutrophils. The evaluation of hnRNPs distributions could predict prognosis of cancer patients. These findings provide novel evidence for the investigation of hnRNPs in the development and therapy of cancer in the future.

## CONFLICT OF INTEREST

All of the authors declare that there is no conflict of interest.

## AUTHOR CONTRIBUTION

**Hao Li:** Formal analysis (equal); Writing‐original draft (equal). **Jingwei Liu:** Formal analysis (equal); Writing‐original draft (equal). **Shixuan Shen:** Investigation (lead); Methodology (lead). **Di Dai:** Validation (equal); Visualization (equal). **Shitong Cheng:** Data curation (equal); Investigation (equal). **Xiaolong Dong:** Formal analysis (supporting); Visualization (supporting). **Liping Sun:** Investigation (equal); Writing‐review & editing (equal). **Xiaolin Guo:** Project administration (equal); Writing‐review & editing (equal).

## Supporting information

Table S1

Table S2

## Data Availability

All of the data in this article were used the TCGA datasets (https://www.cancer.gov/about‐nci/organization/ccg/research/structural‐genomics/tcga).
